# Clinical decision support menu for reducing unnecessary urine cultures

**DOI:** 10.1017/ash.2024.47

**Published:** 2024-05-27

**Authors:** Erik Stensgard, Bobbie Masoud, Amy Gravely, Dimitri Drekonja

**Affiliations:** 1 Minneapolis Veterans Affairs Health Care System, Minneapolis, MN, USA; 2 University of Minnesota Medical School, Minneapolis, MN, USA

## Abstract

A clinical decision support menu requiring selection of urine culture (UC) indication within outpatient and inpatient settings of a large integrated health care system significantly reduced UC sample collection. Clear documentation of selected UC indication in most post-intervention cases affirms its potential as an effective diagnostic stewardship strategy.

## Introduction

Asymptomatic bacteriuria (ASB) is a typically benign condition where bacteria are present in the urine without symptoms of a urinary tract infection (dysuria, increased urinary frequency, or urgency).^1^ Antimicrobial treatment of ASB is not beneficial except in pregnant women or those undergoing invasive urologic procedures.^2^ Unnecessary treatment of ASB is an important driver of inappropriate antimicrobial use, leading to antimicrobial resistance, *Clostridioides difficile* infection, adverse drug events, and increased costs.^3^ ASB is often inappropriately treated and strong evidence supports not obtaining urine cultures (UCs) from asymptomatic patients.^2,4^

Diagnostic stewardship, including conditional reflex to culture and clinical decision support (CDS), is an established method for reducing inappropriate UCs and subsequent antimicrobial days of therapy.^5–8^ A limitation of conditional reflex to culture is UCs may be obtained for asymptomatic patients who have an abnormal urinalysis without symptoms of urinary tract infection. Diagnostic stewardship with CDS interventions that provide clinical guidance and require selection of an indication at the point of UC order entry may reduce inappropriate UCs more than other methods alone by encouraging the clinician to critically assess the need for a UC. This type of CDS intervention has been effective in decreasing UCs in limited settings including inpatients with indwelling catheters or within a single outpatient clinic.^7,8^ To reduce unnecessary UCs in all settings at the Minneapolis Veterans Affairs Health Care System (MVAHCS), clinicians placing UC orders within the electronic medical record (EMR), known as the Computerized Patient Record System (CPRS), were directed to a UC CDS menu, providing clinical guidance and requiring selection of a single indication to place the order.

## Methods

The study population included all veterans receiving care within the MVAHCS. MVAHCS has a patient population of 88,466 and includes a 200-bed medical center in Minneapolis, Minnesota along with 13 community-based outpatient clinics within Minnesota and Wisconsin. The UC CDS menu was installed in MVAHCS’s EMR in September 2020. The EMR implementation process involved creating ten UC laboratory “quick orders” and placing them within a UC CDS menu. These “quick orders” are EMR items containing information on the selected indication and instructions for health care personal to obtain a urine sample for culture from the patient. As part of the installation, when placing a UC order within the EMR, providers were directed to the UC CDS menu and required to select a single indication. A small number of UC ordering pathways were not routed to the UC CDS menu. These were for ordering UCs in extenuating circumstances. Patient bed days were collected as a measure of inpatient volume, and clinic visits were collected as a measure of outpatient volume. A chart review was conducted on a random sample of UCs during the post-intervention study period to determine the degree and accuracy of UC indication documentation within the EMR. Documentation of indication within the patient’s chart was used to determine UC appropriateness.

The study period was 16 months pre-implementation (September 1, 2018–December 31, 2019) and 16 months post-implementation (September 1, 2020–December 31, 2021). The period from January 1, 2020, to August 31, 2020, was excluded due to atypical care patterns during the COVID-19 pandemic. Data were included for patients receiving care at the MVAHCS who had a urine sample collected for culture during the study period. UC information and clinic visit count were acquired from the Corporate Data Warehouse (CDW), the Veteran Affairs’ (VA) data repository. Patient bed days were derived from a CDW dashboard developed by the Iowa City VA Health Care System.^9^ Documentation of UC indication post-intervention was evaluated by reviewing a random sample of 50 UCs for evidence of selected indication documented within the EMR. The review was conducted by a health science specialist under the supervision of an infectious diseases physician. Readmissions, infection rates, and mortality data were not systematically collected for this study. This project was reviewed by the MVAHCS institutional review board and determined to fall under quality improvement and thus not requiring their oversight.

Analyses were conducted first on the outpatient UC samples collected per 1000 clinic visits then separately on inpatient UC samples collected per 1000 patient bed days. We tested the difference in intercepts and slopes before and after the intervention using interrupted time-series analysis conducted in SAS 9.4 software (SAS Institute, Cary, NC). Two-sample *t*-test was used to compare pre- versus post-intervention monthly mean separately for UC sample collection count, patient bed days, and clinic visits.

## Results

**Figure 1.**
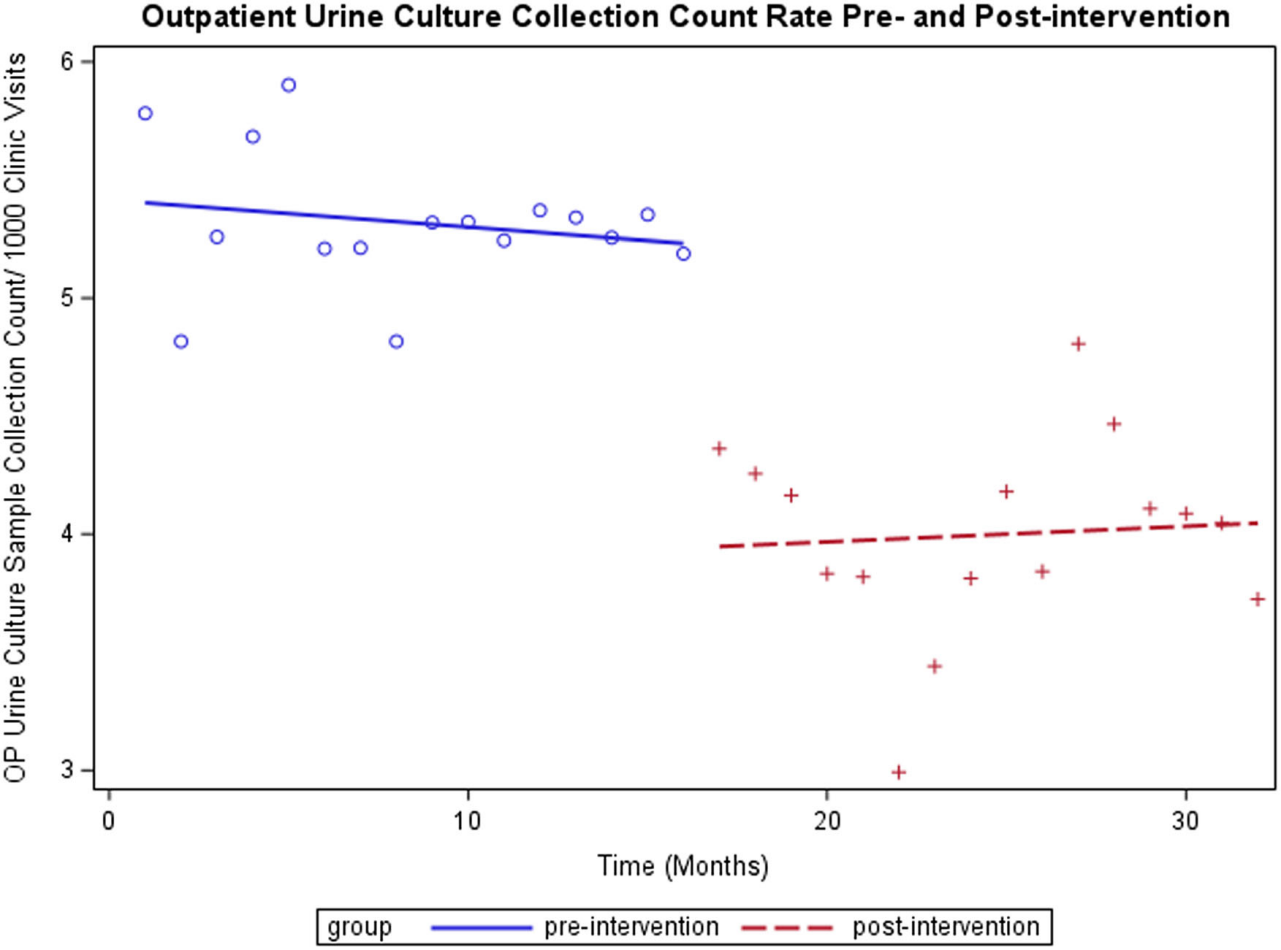
Outpatient (OP) interrupted time-series analysis.

**Figure 2.**
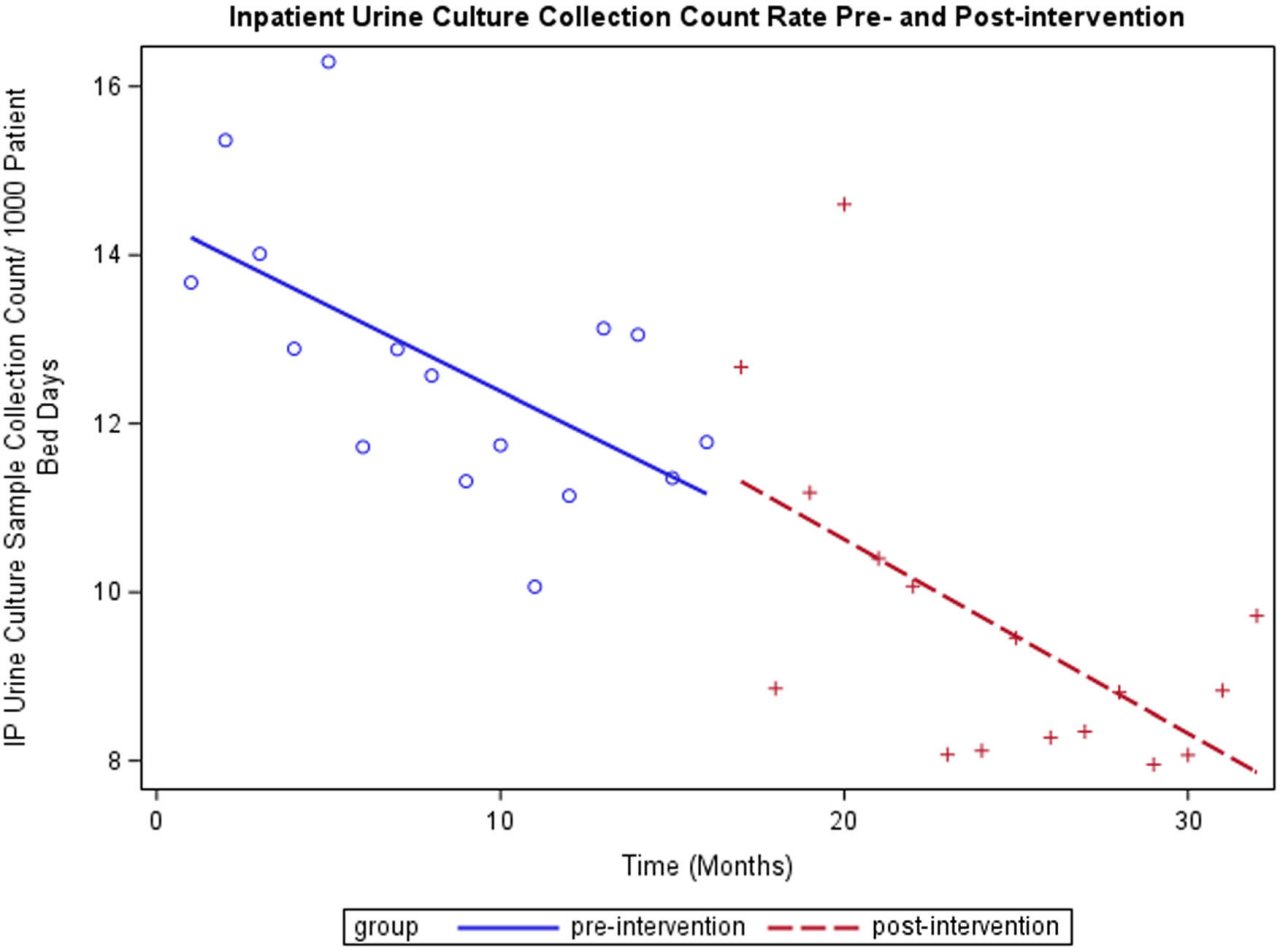
Inpatient (IP) interrupted time-series analysis.

For outpatient UC samples collected per 1000 clinic visits, the intervention was associated with a reduction in UCs from an average of 5.22 per 1000 outpatient visits to 3.94 (*P* < .0001) (Figure 1). The temporal trends (slopes) pre- and post-intervention were not significantly different (*P* = .53). For inpatient, the intervention was not associated with a significant overall change, with 10.96 inpatient UC samples collected per 1000 patient bed days pre-intervention and 11.54 post-intervention (*P* = .59) (Figure 2). The temporal trend in UCs was not significantly different pre- and post-intervention *P* = .80), with a clear trend of decreasing UCs before and after the intervention. Overall, total UCs were reduced by 26% (12190 pre- and 8996 post-intervention). There was a significant reduction in mean monthly UC sample collection count from pre- (mean = 762) versus post-intervention (mean = 562) (*P* < .0001). Patient bed days and clinic visits were not significantly changed.

Post-intervention, most UCs originated from the UC CDS menu (8103, 90%), while the remaining UCs were ordered from other pathways (893, 10%). The most common indication selected was dysuria, frequency, and urgency (4049, 45%) followed by fever or sepsis (1230, 14%) and preoperative urologic screening (1056, 12%). An EMR chart review of post-intervention cases showed that 35 out of 50 (70%) randomly selected UCs had clear documentation of the selected indication.

## Discussion

Implementation of the UC CDS menu resulted in a sizable and statistically significant reduction in total UC sample collection count. There was a significant reduction in monthly outpatient UCs, whereas inpatient UCs were trending downward pre-intervention and continued post-intervention. We suspect the inpatient data reflects a prior robust effort to decrease UCs via provider education, which was not present in the outpatient setting.^10^ This CDS intervention was an effort to continue the prior educational intervention without dedicated staffing and expand it to all care settings. Most of the post-intervention UCs had clear evidence of the selected indication recorded in the EMR. This suggests that the decrease in UCs was due to preventing inappropriate UCs, and not clinicians simply selecting an appropriate indication, regardless of the patient’s actual symptoms. Limitations of this study include results were from a single health care system using retrospective data collection and the intervention was implemented within only one EMR, CPRS – VA’s legacy EMR, which may have a different ordering interface from other widely used EMRs.

Since this intervention was made, five additional VA health care systems have installed the same UC CDS menu within their own EMR with similar reductions in UCs. The UC CDS menu at the MVAHCS was associated with a significant and meaningful reduction in UCs, especially within outpatient settings. It is an effective diagnostic stewardship strategy with demonstrated scalability that has potential for broad implementation within other health care systems.
